# Supplementary material for: Screening frost-tolerant sunflower hybrids: integrating physiological traits and electrolyte leakage analysis
Source: PeerJ. 2025 Nov 7;13:e20282. doi: 10.7717/peerj.20282 (PMC12599371; doi:10.7717/peerj.20282)
Supplement: Supplemental Information 2 [file peerj-13-20282-s002.docx]

**V4 PERIOD**

**SPAD**

| **Source** | **DF** | **Sum of Squares** | **Mean Square** | **F Ratio** |
| --- | --- | --- | --- | --- |
| Model | 27 | 840.02170 | 31.1119 | 21.3317 |
| Error | 84 | 122.51250 | 1.4585 | **Prob > F** |
| C. Total | 111 | 962.53420 |  | <.0001* |

| **Source** | **Nparm** | **DF** | **Sum of Squares** | **F Ratio** | **Prob > F** |
| --- | --- | --- | --- | --- | --- |
| Çeşit | 13 | 13 | 339.06795 | 17.8831 | <.0001* |
| Soğuk | 1 | 1 | 48.76080 | 33.4326 | <.0001* |
| Çeşit*Soğuk | 13 | 13 | 452.19295 | 23.8495 | <.0001* |

| **Level** |  |  |  |  |  |  |  | **Least Sq Mean** |
| --- | --- | --- | --- | --- | --- | --- | --- | --- |
| 6 | A |  |  |  |  |  |  | 41.337500 |
| 9 |  | B |  |  |  |  |  | 40.037500 |
| 7 |  |  | C |  |  |  |  | 37.462500 |
| 1 |  |  | C | D |  |  |  | 37.425000 |
| 10 |  |  | C | D | E |  |  | 37.325000 |
| 13 |  |  | C | D | E |  |  | 37.225000 |
| 12 |  |  | C | D | E | F |  | 36.462500 |
| 5 |  |  | C | D | E | F |  | 36.312500 |
| 14 |  |  |  | D | E | F |  | 36.225000 |
| 11 |  |  |  |  | E | F |  | 36.125000 |
| 2 |  |  |  |  |  | F |  | 35.962500 |
| 3 |  |  |  |  |  | F |  | 35.787500 |
| 8 |  |  |  |  |  | F |  | 35.750000 |
| 4 |  |  |  |  |  |  | G | 34.250000 |

| **Level** |  |  | **Least Sq Mean** |
| --- | --- | --- | --- |
| 2 | A |  | 37.637500 |
| 1 |  | B | 36.317857 |

| **Level** |  |  |  |  |  |  |  |  |  |  |  |  |  |  |  | **Least Sq Mean** |
| --- | --- | --- | --- | --- | --- | --- | --- | --- | --- | --- | --- | --- | --- | --- | --- | --- |
| 6,1 | A |  |  |  |  |  |  |  |  |  |  |  |  |  |  | 45.450000 |
| 10,2 |  | B |  |  |  |  |  |  |  |  |  |  |  |  |  | 41.200000 |
| 9,2 |  | B |  |  |  |  |  |  |  |  |  |  |  |  |  | 41.025000 |
| 1,2 |  | B | C |  |  |  |  |  |  |  |  |  |  |  |  | 40.600000 |
| 9,1 |  |  | C | D |  |  |  |  |  |  |  |  |  |  |  | 39.050000 |
| 7,2 |  |  | C | D | E |  |  |  |  |  |  |  |  |  |  | 39.025000 |
| 11,2 |  |  |  | D | E | F |  |  |  |  |  |  |  |  |  | 38.900000 |
| 14,2 |  |  |  | D | E | F | G |  |  |  |  |  |  |  |  | 38.100000 |
| 12,2 |  |  |  | D | E | F | G | H |  |  |  |  |  |  |  | 37.875000 |
| 13,1 |  |  |  | D | E | F | G | H |  |  |  |  |  |  |  | 37.850000 |
| 3,1 |  |  |  |  | E | F | G | H | I |  |  |  |  |  |  | 37.350000 |
| 6,2 |  |  |  |  |  | F | G | H | I |  |  |  |  |  |  | 37.225000 |
| 5,1 |  |  |  |  |  |  | G | H | I | J |  |  |  |  |  | 36.800000 |
| 2,2 |  |  |  |  |  |  | G | H | I | J |  |  |  |  |  | 36.775000 |
| 13,2 |  |  |  |  |  |  | G | H | I | J | K |  |  |  |  | 36.600000 |
| 8,1 |  |  |  |  |  |  |  | H | I | J | K |  |  |  |  | 36.250000 |
| 7,1 |  |  |  |  |  |  |  |  | I | J | K | L |  |  |  | 35.900000 |
| 5,2 |  |  |  |  |  |  |  |  | I | J | K | L | M |  |  | 35.825000 |
| 8,2 |  |  |  |  |  |  |  |  |  | J | K | L | M |  |  | 35.250000 |
| 2,1 |  |  |  |  |  |  |  |  |  | J | K | L | M |  |  | 35.150000 |
| 12,1 |  |  |  |  |  |  |  |  |  |  | K | L | M | N |  | 35.050000 |
| 14,1 |  |  |  |  |  |  |  |  |  |  |  | L | M | N | O | 34.350000 |
| 4,2 |  |  |  |  |  |  |  |  |  |  |  | L | M | N | O | 34.300000 |
| 1,1 |  |  |  |  |  |  |  |  |  |  |  | L | M | N | O | 34.250000 |
| 3,2 |  |  |  |  |  |  |  |  |  |  |  | L | M | N | O | 34.225000 |
| 4,1 |  |  |  |  |  |  |  |  |  |  |  |  | M | N | O | 34.200000 |
| 10,1 |  |  |  |  |  |  |  |  |  |  |  |  |  | N | O | 33.450000 |
| 11,1 |  |  |  |  |  |  |  |  |  |  |  |  |  |  | O | 33.350000 |

**LEAF TEMPERATURE**

| **Source** | **DF** | **Sum of Squares** | **Mean Square** | **F Ratio** |
| --- | --- | --- | --- | --- |
| Model | 27 | 545.16107 | 20.1912 | 104.7595 |
| Error | 84 | 16.19000 | 0.1927 | **Prob > F** |
| C. Total | 111 | 561.35107 |  | <.0001* |

| **Source** | **Nparm** | **DF** | **Sum of Squares** | **F Ratio** | **Prob > F** |
| --- | --- | --- | --- | --- | --- |
| Çeşit | 13 | 13 | 411.85107 | 164.3725 | <.0001* |
| Soğuk | 1 | 1 | 1.80036 | 9.3410 | 0.0030* |
| Çeşit*Soğuk | 13 | 13 | 131.50964 | 52.4864 | <.0001* |

| **Level** |  |  |  |  |  |  |  |  |  | **Least Sq Mean** |
| --- | --- | --- | --- | --- | --- | --- | --- | --- | --- | --- |
| 6 | A |  |  |  |  |  |  |  |  | 26.675000 |
| 4 |  | B |  |  |  |  |  |  |  | 26.162500 |
| 10 |  |  | C |  |  |  |  |  |  | 23.125000 |
| 2 |  |  | C |  |  |  |  |  |  | 22.875000 |
| 3 |  |  |  | D |  |  |  |  |  | 22.162500 |
| 9 |  |  |  | D |  |  |  |  |  | 22.150000 |
| 5 |  |  |  | D | E |  |  |  |  | 21.975000 |
| 8 |  |  |  | D | E |  |  |  |  | 21.862500 |
| 7 |  |  |  |  | E | F |  |  |  | 21.587500 |
| 12 |  |  |  |  |  | F | G |  |  | 21.237500 |
| 11 |  |  |  |  |  |  | G | H |  | 20.950000 |
| 1 |  |  |  |  |  |  |  | H |  | 20.787500 |
| 14 |  |  |  |  |  |  |  | H |  | 20.612500 |
| 13 |  |  |  |  |  |  |  |  | I | 19.562500 |

| **Level** |  |  | **Least Sq Mean** |
| --- | --- | --- | --- |
| 2 | A |  | 22.392857 |
| 1 |  | B | 22.139286 |

| **Level** |  |  |  |  |  |  |  |  |  |  |  |  |  | **Least Sq Mean** |
| --- | --- | --- | --- | --- | --- | --- | --- | --- | --- | --- | --- | --- | --- | --- |
| 6,1 | A |  |  |  |  |  |  |  |  |  |  |  |  | 26.900000 |
| 6,2 | A | B |  |  |  |  |  |  |  |  |  |  |  | 26.450000 |
| 4,2 |  | B |  |  |  |  |  |  |  |  |  |  |  | 26.175000 |
| 4,1 |  | B |  |  |  |  |  |  |  |  |  |  |  | 26.150000 |
| 10,1 |  |  | C |  |  |  |  |  |  |  |  |  |  | 24.400000 |
| 2,2 |  |  | C |  |  |  |  |  |  |  |  |  |  | 24.350000 |
| 3,2 |  |  | C | D |  |  |  |  |  |  |  |  |  | 23.825000 |
| 9,1 |  |  |  | D | E |  |  |  |  |  |  |  |  | 23.500000 |
| 8,1 |  |  |  |  | E |  |  |  |  |  |  |  |  | 23.150000 |
| 5,2 |  |  |  |  | E |  |  |  |  |  |  |  |  | 23.150000 |
| 7,1 |  |  |  |  | E | F |  |  |  |  |  |  |  | 22.950000 |
| 1,2 |  |  |  |  |  | F | G |  |  |  |  |  |  | 22.375000 |
| 12,2 |  |  |  |  |  |  | G | H |  |  |  |  |  | 21.875000 |
| 10,2 |  |  |  |  |  |  | G | H |  |  |  |  |  | 21.850000 |
| 2,1 |  |  |  |  |  |  |  | H | I |  |  |  |  | 21.400000 |
| 11,1 |  |  |  |  |  |  |  |  | I | J |  |  |  | 21.000000 |
| 11,2 |  |  |  |  |  |  |  |  | I | J |  |  |  | 20.900000 |
| 5,1 |  |  |  |  |  |  |  |  | I | J | K |  |  | 20.800000 |
| 9,2 |  |  |  |  |  |  |  |  | I | J | K |  |  | 20.800000 |
| 14,2 |  |  |  |  |  |  |  |  |  | J | K | L |  | 20.775000 |
| 12,1 |  |  |  |  |  |  |  |  |  | J | K | L |  | 20.600000 |
| 8,2 |  |  |  |  |  |  |  |  |  | J | K | L |  | 20.575000 |
| 3,1 |  |  |  |  |  |  |  |  |  | J | K | L |  | 20.500000 |
| 14,1 |  |  |  |  |  |  |  |  |  | J | K | L |  | 20.450000 |
| 7,2 |  |  |  |  |  |  |  |  |  |  | K | L |  | 20.225000 |
| 13,2 |  |  |  |  |  |  |  |  |  |  |  | L |  | 20.175000 |
| 1,1 |  |  |  |  |  |  |  |  |  |  |  |  | M | 19.200000 |
| 13,1 |  |  |  |  |  |  |  |  |  |  |  |  | M | 18.950000 |

**RELATIVE WATER CONTENT**

| **Source** | **DF** | **Sum of Squares** | **Mean Square** | **F Ratio** |
| --- | --- | --- | --- | --- |
| Model | 27 | 9335.340 | 345.753 | 20.4964 |
| Error | 84 | 1416.998 | 16.869 | **Prob > F** |
| C. Total | 111 | 10752.338 |  | <.0001* |

| **Source** | **Nparm** | **DF** | **Sum of Squares** | **F Ratio** | **Prob > F** |
| --- | --- | --- | --- | --- | --- |
| Çeşit | 13 | 13 | 7483.8640 | 34.1266 | <.0001* |
| Soğuk | 1 | 1 | 102.4144 | 6.0712 | 0.0158* |
| Çeşit*Soğuk | 13 | 13 | 1749.0619 | 7.9758 | <.0001* |

| **Level** |  |  |  |  |  |  |  |  | **Least Sq Mean** |
| --- | --- | --- | --- | --- | --- | --- | --- | --- | --- |
| 3 | A |  |  |  |  |  |  |  | 105.97500 |
| 12 |  | B |  |  |  |  |  |  | 97.51250 |
| 2 |  |  | C |  |  |  |  |  | 92.65000 |
| 9 |  |  | C | D |  |  |  |  | 91.05000 |
| 1 |  |  |  | D | E |  |  |  | 88.28750 |
| 11 |  |  |  | D | E | F |  |  | 87.03750 |
| 8 |  |  |  |  | E | F | G |  | 86.56250 |
| 10 |  |  |  |  | E | F | G |  | 86.05000 |
| 5 |  |  |  |  | E | F | G |  | 85.68750 |
| 7 |  |  |  |  | E | F | G |  | 84.38750 |
| 14 |  |  |  |  |  | F | G |  | 83.21250 |
| 13 |  |  |  |  |  |  | G |  | 82.88750 |
| 6 |  |  |  |  |  |  |  | H | 75.45000 |
| 4 |  |  |  |  |  |  |  | H | 71.48750 |

| **Level** |  |  | **Least Sq Mean** |
| --- | --- | --- | --- |
| 2 | A |  | 87.973214 |
| 1 |  | B | 86.060714 |

| **Level** |  |  |  |  |  |  |  |  |  |  |  |  |  | **Least Sq Mean** |
| --- | --- | --- | --- | --- | --- | --- | --- | --- | --- | --- | --- | --- | --- | --- |
| 3,1 | A |  |  |  |  |  |  |  |  |  |  |  |  | 113.00000 |
| 12,2 |  | B |  |  |  |  |  |  |  |  |  |  |  | 101.77500 |
| 3,2 |  | B | C |  |  |  |  |  |  |  |  |  |  | 98.95000 |
| 2,1 |  | B | C | D |  |  |  |  |  |  |  |  |  | 96.15000 |
| 5,2 |  |  | C | D |  |  |  |  |  |  |  |  |  | 95.77500 |
| 12,1 |  |  | C | D | E |  |  |  |  |  |  |  |  | 93.25000 |
| 9,2 |  |  |  | D | E | F |  |  |  |  |  |  |  | 91.50000 |
| 9,1 |  |  |  | D | E | F |  |  |  |  |  |  |  | 90.60000 |
| 2,2 |  |  |  |  | E | F | G |  |  |  |  |  |  | 89.15000 |
| 8,1 |  |  |  |  | E | F | G |  |  |  |  |  |  | 89.10000 |
| 1,2 |  |  |  |  | E | F | G |  |  |  |  |  |  | 88.82500 |
| 11,1 |  |  |  |  | E | F | G |  |  |  |  |  |  | 88.30000 |
| 1,1 |  |  |  |  | E | F | G |  |  |  |  |  |  | 87.75000 |
| 14,2 |  |  |  |  | E | F | G |  |  |  |  |  |  | 87.67500 |
| 10,2 |  |  |  |  |  | F | G | H |  |  |  |  |  | 86.35000 |
| 11,2 |  |  |  |  |  | F | G | H |  |  |  |  |  | 85.77500 |
| 10,1 |  |  |  |  |  | F | G | H |  |  |  |  |  | 85.75000 |
| 7,2 |  |  |  |  |  |  | G | H | I |  |  |  |  | 84.42500 |
| 7,1 |  |  |  |  |  |  | G | H | I |  |  |  |  | 84.35000 |
| 13,2 |  |  |  |  |  |  | G | H | I |  |  |  |  | 84.27500 |
| 8,2 |  |  |  |  |  |  | G | H | I |  |  |  |  | 84.02500 |
| 13,1 |  |  |  |  |  |  |  | H | I | J |  |  |  | 81.50000 |
| 14,1 |  |  |  |  |  |  |  |  | I | J | K |  |  | 78.75000 |
| 6,2 |  |  |  |  |  |  |  |  |  | J | K | L |  | 78.15000 |
| 5,1 |  |  |  |  |  |  |  |  |  |  | K | L |  | 75.60000 |
| 4,2 |  |  |  |  |  |  |  |  |  |  | K | L |  | 74.97500 |
| 6,1 |  |  |  |  |  |  |  |  |  |  |  | L | M | 72.75000 |
| 4,1 |  |  |  |  |  |  |  |  |  |  |  |  | M | 68.00000 |

**ELECTROLYTE LEAKAGE**

| **Source** | **DF** | **Sum of Squares** | **Mean Square** | **F Ratio** |
| --- | --- | --- | --- | --- |
| Model | 27 | 17976.612 | 665.800 | 74.4530 |
| Error | 84 | 751.175 | 8.943 | **Prob > F** |
| C. Total | 111 | 18727.787 |  | <.0001* |

| **Source** | **Nparm** | **DF** | **Sum of Squares** | **F Ratio** | **Prob > F** |
| --- | --- | --- | --- | --- | --- |
| Çeşit | 13 | 13 | 9639.9296 | 82.9218 | <.0001* |
| Soğuk | 1 | 1 | 4628.5714 | 517.5891 | <.0001* |
| Çeşit*Soğuk | 13 | 13 | 3708.1111 | 31.8968 | <.0001* |

| **Level** |  |  |  |  | **Least Sq Mean** |
| --- | --- | --- | --- | --- | --- |
| 13 | A |  |  |  | 50.112500 |
| 11 | A |  |  |  | 50.050000 |
| 1 | A |  |  |  | 49.787500 |
| 14 | A |  |  |  | 49.750000 |
| 10 | A |  |  |  | 45.737500 |
| 9 |  | B |  |  | 38.462500 |
| 6 |  | B |  |  | 37.675000 |
| 12 |  | B |  |  | 37.662500 |
| 7 |  | B | C |  | 35.150000 |
| 4 |  |  | C |  | 31.112500 |
| 8 |  |  | C |  | 30.800000 |
| 2 |  |  | C |  | 30.750000 |
| 5 |  |  |  | D | 24.450000 |
| 3 |  |  |  | D | 22.750000 |

| **Level** |  |  | **Least Sq Mean** |
| --- | --- | --- | --- |
| 2 | A |  | 44.589286 |
| 1 |  | B | 31.732143 |

| **Level** |  |  |  |  |  |  |  |  |  |  |  |  |  | **Least Sq Mean** |
| --- | --- | --- | --- | --- | --- | --- | --- | --- | --- | --- | --- | --- | --- | --- |
| 10,2 | A |  |  |  |  |  |  |  |  |  |  |  |  | 62.625000 |
| 14,2 | A |  |  |  |  |  |  |  |  |  |  |  |  | 60.050000 |
| 11,2 | A |  |  |  |  |  |  |  |  |  |  |  |  | 59.500000 |
| 13,2 | A |  |  |  |  |  |  |  |  |  |  |  |  | 58.925000 |
| 9,2 | A | B |  |  |  |  |  |  |  |  |  |  |  | 54.875000 |
| 1,1 |  | B | C |  |  |  |  |  |  |  |  |  |  | 49.900000 |
| 1,2 |  | B | C |  |  |  |  |  |  |  |  |  |  | 49.675000 |
| 12,2 |  | B | C | D |  |  |  |  |  |  |  |  |  | 47.625000 |
| 7,2 |  |  | C | D | E |  |  |  |  |  |  |  |  | 43.050000 |
| 13,1 |  |  |  | D | E |  |  |  |  |  |  |  |  | 41.300000 |
| 11,1 |  |  |  | D | E | F |  |  |  |  |  |  |  | 40.600000 |
| 6,2 |  |  |  | D | E | F | G |  |  |  |  |  |  | 39.500000 |
| 14,1 |  |  |  |  | E | F | G |  |  |  |  |  |  | 39.450000 |
| 2,2 |  |  |  |  | E | F | G | H |  |  |  |  |  | 37.100000 |
| 6,1 |  |  |  |  | E | F | G | H | I |  |  |  |  | 35.850000 |
| 4,2 |  |  |  |  |  | F | G | H | I | J |  |  |  | 32.825000 |
| 8,2 |  |  |  |  |  |  | G | H | I | J | K |  |  | 32.100000 |
| 8,1 |  |  |  |  |  |  |  | H | I | J | K | L |  | 29.500000 |
| 4,1 |  |  |  |  |  |  |  | H | I | J | K | L |  | 29.400000 |
| 10,1 |  |  |  |  |  |  |  |  | I | J | K | L | M | 28.850000 |
| 12,1 |  |  |  |  |  |  |  |  |  | J | K | L | M | 27.700000 |
| 7,1 |  |  |  |  |  |  |  |  |  | J | K | L | M | 27.250000 |
| 5,2 |  |  |  |  |  |  |  |  |  | J | K | L | M | 25.500000 |
| 3,1 |  |  |  |  |  |  |  |  |  |  | K | L | M | 24.600000 |
| 2,1 |  |  |  |  |  |  |  |  |  |  | K | L | M | 24.400000 |
| 5,1 |  |  |  |  |  |  |  |  |  |  |  | L | M | 23.400000 |
| 9,1 |  |  |  |  |  |  |  |  |  |  |  | L | M | 22.050000 |
| 3,2 |  |  |  |  |  |  |  |  |  |  |  |  | M | 20.900000 |
