# Supplementary material for: Screening frost-tolerant sunflower hybrids: integrating physiological traits and electrolyte leakage analysis
Source: PeerJ. 2025 Nov 7;13:e20282. doi: 10.7717/peerj.20282 (PMC12599371; doi:10.7717/peerj.20282)
Supplement: Supplemental Information 3 [file peerj-13-20282-s003.docx]

**V2 PERIOD**

**SPAD**

| **Source** | **DF** | **Sum of Squares** | **Mean Square** | **F Ratio** |
| --- | --- | --- | --- | --- |
| Model | 27 | 589.33313 | 21.8272 | 7.0518 |
| Error | 84 | 260.00250 | 3.0953 | **Prob > F** |
| C. Total | 111 | 849.33563 |  | <.0001* |

| **Source** | **Nparm** | **DF** | **Sum of Squares** | **F Ratio** | **Prob > F** |
| --- | --- | --- | --- | --- | --- |
| Çeşit | 13 | 13 | 324.58938 | 8.0666 | <.0001* |
| Soğuk | 1 | 1 | 0.03223 | 0.0104 | 0.9190 |
| Çeşit*Soğuk | 13 | 13 | 264.71152 | 6.5786 | <.0001* |

| **Level** |  |  |  |  | **Least Sq Mean** |
| --- | --- | --- | --- | --- | --- |
| 2 | A |  |  |  | 39.212500 |
| 6 | A | B |  |  | 38.850000 |
| 9 | A | B |  |  | 38.562500 |
| 3 | A | B |  |  | 38.525000 |
| 10 | A | B |  |  | 38.350000 |
| 12 | A | B |  |  | 38.275000 |
| 1 | A | B |  |  | 37.962500 |
| 13 | A | B |  |  | 37.950000 |
| 5 | A | B |  |  | 37.550000 |
| 8 |  | B |  |  | 37.237500 |
| 7 |  | B |  |  | 37.137500 |
| 14 |  |  | C |  | 35.137500 |
| 4 |  |  | C |  | 35.125000 |
| 11 |  |  |  | D | 32.937500 |

| **Level** | **Least Sq Mean** | **Std Error** | **Mean** |
| --- | --- | --- | --- |
| 1 | 37.360714 | 0.23510134 | 37.3607 |
| 2 | 37.326786 | 0.23510134 | 37.3268 |

| **Level** |  |  |  |  |  |  |  |  |  |  |  |  | **Least Sq Mean** |
| --- | --- | --- | --- | --- | --- | --- | --- | --- | --- | --- | --- | --- | --- |
| 3,2 | A |  |  |  |  |  |  |  |  |  |  |  | 41.450000 |
| 2,2 | A | B |  |  |  |  |  |  |  |  |  |  | 41.225000 |
| 6,1 | A | B | C |  |  |  |  |  |  |  |  |  | 40.200000 |
| 13,2 | A | B | C |  |  |  |  |  |  |  |  |  | 40.000000 |
| 12,1 | A | B | C | D |  |  |  |  |  |  |  |  | 39.600000 |
| 9,2 | A | B | C | D | E |  |  |  |  |  |  |  | 39.325000 |
| 10,1 |  | B | C | D | E | F |  |  |  |  |  |  | 38.900000 |
| 5,1 |  |  | C | D | E | F | G |  |  |  |  |  | 38.700000 |
| 1,2 |  |  | C | D | E | F | G | H |  |  |  |  | 38.075000 |
| 1,1 |  |  | C | D | E | F | G | H | I |  |  |  | 37.850000 |
| 9,1 |  |  | C | D | E | F | G | H | I |  |  |  | 37.800000 |
| 10,2 |  |  | C | D | E | F | G | H | I |  |  |  | 37.800000 |
| 6,2 |  |  |  | D | E | F | G | H | I |  |  |  | 37.500000 |
| 7,2 |  |  |  | D | E | F | G | H | I |  |  |  | 37.475000 |
| 8,1 |  |  |  | D | E | F | G | H | I |  |  |  | 37.400000 |
| 2,1 |  |  |  | D | E | F | G | H | I |  |  |  | 37.200000 |
| 8,2 |  |  |  |  | E | F | G | H | I | J |  |  | 37.075000 |
| 12,2 |  |  |  |  | E | F | G | H | I | J |  |  | 36.950000 |
| 14,1 |  |  |  |  |  | F | G | H | I | J |  |  | 36.800000 |
| 7,1 |  |  |  |  |  | F | G | H | I | J |  |  | 36.800000 |
| 5,2 |  |  |  |  |  |  | G | H | I | J |  |  | 36.400000 |
| 13,1 |  |  |  |  |  |  |  | H | I | J | K |  | 35.900000 |
| 11,1 |  |  |  |  |  |  |  | H | I | J | K |  | 35.650000 |
| 3,1 |  |  |  |  |  |  |  |  | I | J | K |  | 35.600000 |
| 4,2 |  |  |  |  |  |  |  |  | I | J | K |  | 35.600000 |
| 4,1 |  |  |  |  |  |  |  |  |  | J | K |  | 34.650000 |
| 14,2 |  |  |  |  |  |  |  |  |  |  | K |  | 33.475000 |
| 11,2 |  |  |  |  |  |  |  |  |  |  |  | L | 30.225000 |

**LEAF TEMPERATURE**

| **Source** | **DF** | **Sum of Squares** | **Mean Square** | **F Ratio** |
| --- | --- | --- | --- | --- |
| Model | 27 | 429.23170 | 15.8975 | 132.4461 |
| Error | 84 | 10.08250 | 0.1200 | **Prob > F** |
| C. Total | 111 | 439.31420 |  | <.0001* |

| **Source** | **Nparm** | **DF** | **Sum of Squares** | **F Ratio** | **Prob > F** |
| --- | --- | --- | --- | --- | --- |
| Çeşit | 13 | 13 | 58.93295 | 37.7682 | <.0001* |
| Soğuk | 1 | 1 | 327.43080 | 2727.913 | <.0001* |
| Çeşit*Soğuk | 13 | 13 | 42.86795 | 27.4726 | <.0001* |

| **Level** |  |  |  |  |  | **Least Sq Mean** |
| --- | --- | --- | --- | --- | --- | --- |
| 13 | A |  |  |  |  | 23.387500 |
| 10 |  | B |  |  |  | 22.975000 |
| 12 |  | B |  |  |  | 22.975000 |
| 11 |  | B |  |  |  | 22.887500 |
| 14 |  | B |  |  |  | 22.687500 |
| 9 |  |  | C |  |  | 22.275000 |
| 8 |  |  | C |  |  | 22.112500 |
| 7 |  |  | C |  |  | 22.062500 |
| 5 |  |  |  | D |  | 21.587500 |
| 2 |  |  |  | D |  | 21.500000 |
| 1 |  |  |  | D |  | 21.475000 |
| 6 |  |  |  | D |  | 21.400000 |
| 3 |  |  |  | D | E | 21.337500 |
| 4 |  |  |  |  | E | 21.050000 |

| **Level** |  |  | **Least Sq Mean** |
| --- | --- | --- | --- |
| 1 | A |  | 23.832143 |
| 2 |  | B | 20.412500 |

| **Level** |  |  |  |  |  |  |  |  |  | **Least Sq Mean** |
| --- | --- | --- | --- | --- | --- | --- | --- | --- | --- | --- |
| 10,1 | A |  |  |  |  |  |  |  |  | 24.200000 |
| 11,1 | A |  |  |  |  |  |  |  |  | 24.200000 |
| 1,1 | A | B |  |  |  |  |  |  |  | 24.100000 |
| 9,1 | A | B |  |  |  |  |  |  |  | 24.100000 |
| 12,1 | A | B |  |  |  |  |  |  |  | 24.050000 |
| 7,1 | A | B | C |  |  |  |  |  |  | 23.900000 |
| 13,1 | A | B | C |  |  |  |  |  |  | 23.900000 |
| 6,1 | A | B | C |  |  |  |  |  |  | 23.800000 |
| 2,1 | A | B | C | D |  |  |  |  |  | 23.750000 |
| 5,1 | A | B | C | D |  |  |  |  |  | 23.750000 |
| 8,1 |  | B | C | D |  |  |  |  |  | 23.650000 |
| 14,1 |  |  | C | D |  |  |  |  |  | 23.500000 |
| 3,1 |  |  | C | D |  |  |  |  |  | 23.450000 |
| 4,1 |  |  |  | D | E |  |  |  |  | 23.300000 |
| 13,2 |  |  |  |  | E |  |  |  |  | 22.875000 |
| 12,2 |  |  |  |  |  | F |  |  |  | 21.900000 |
| 14,2 |  |  |  |  |  | F |  |  |  | 21.875000 |
| 10,2 |  |  |  |  |  | F |  |  |  | 21.750000 |
| 11,2 |  |  |  |  |  | F |  |  |  | 21.575000 |
| 8,2 |  |  |  |  |  |  | G |  |  | 20.575000 |
| 9,2 |  |  |  |  |  |  | G |  |  | 20.450000 |
| 7,2 |  |  |  |  |  |  | G |  |  | 20.225000 |
| 5,2 |  |  |  |  |  |  |  | H |  | 19.425000 |
| 2,2 |  |  |  |  |  |  |  | H | I | 19.250000 |
| 3,2 |  |  |  |  |  |  |  | H | I | 19.225000 |
| 6,2 |  |  |  |  |  |  |  | H | I | 19.000000 |
| 1,2 |  |  |  |  |  |  |  |  | I | 18.850000 |
| 4,2 |  |  |  |  |  |  |  |  | I | 18.800000 |

**RELATIVE WATER CONTENT**

| **Source** | **DF** | **Sum of Squares** | **Mean Square** | **F Ratio** |
| --- | --- | --- | --- | --- |
| Model | 27 | 1633.5386 | 60.5014 | 7.8935 |
| Error | 84 | 643.8400 | 7.6648 | **Prob > F** |
| C. Total | 111 | 2277.3786 |  | <.0001* |

| **Source** | **Nparm** | **DF** | **Sum of Squares** | **F Ratio** | **Prob > F** |
| --- | --- | --- | --- | --- | --- |
| Çeşit | 13 | 13 | 536.71357 | 5.3864 | <.0001* |
| Soğuk | 1 | 1 | 201.96571 | 26.3499 | <.0001* |
| Çeşit*Soğuk | 13 | 13 | 894.85929 | 8.9808 | <.0001* |

| **Level** |  |  |  |  |  |  | **Least Sq Mean** |
| --- | --- | --- | --- | --- | --- | --- | --- |
| 5 | A |  |  |  |  |  | 83.075000 |
| 6 | A | B |  |  |  |  | 81.250000 |
| 7 |  | B | C |  |  |  | 79.887500 |
| 1 |  | B | C |  |  |  | 79.662500 |
| 11 |  | B | C |  |  |  | 79.412500 |
| 2 |  | B | C |  |  |  | 79.350000 |
| 9 |  | B | C | D |  |  | 78.775000 |
| 3 |  |  | C | D | E |  | 78.425000 |
| 8 |  |  | C | D | E |  | 78.137500 |
| 14 |  |  | C | D | E |  | 78.000000 |
| 10 |  |  | C | D | E |  | 77.400000 |
| 4 |  |  |  | D | E | F | 76.475000 |
| 12 |  |  |  |  | E | F | 75.925000 |
| 13 |  |  |  |  |  | F | 73.875000 |

| **Level** |  |  | **Least Sq Mean** |
| --- | --- | --- | --- |
| 1 | A |  | 79.889286 |
| 2 |  | B | 77.203571 |

| **Level** |  |  |  |  |  |  |  |  |  |  |  |  |  | **Least Sq Mean** |
| --- | --- | --- | --- | --- | --- | --- | --- | --- | --- | --- | --- | --- | --- | --- |
| 2,1 | A |  |  |  |  |  |  |  |  |  |  |  |  | 86.150000 |
| 6,1 | A | B |  |  |  |  |  |  |  |  |  |  |  | 85.600000 |
| 5,1 | A | B |  |  |  |  |  |  |  |  |  |  |  | 85.200000 |
| 9,2 | A | B | C |  |  |  |  |  |  |  |  |  |  | 83.600000 |
| 11,1 |  | B | C | D |  |  |  |  |  |  |  |  |  | 82.000000 |
| 5,2 |  |  | C | D | E |  |  |  |  |  |  |  |  | 80.950000 |
| 10,1 |  |  | C | D | E | F |  |  |  |  |  |  |  | 80.550000 |
| 4,1 |  |  | C | D | E | F | G |  |  |  |  |  |  | 79.950000 |
| 7,2 |  |  | C | D | E | F | G |  |  |  |  |  |  | 79.925000 |
| 7,1 |  |  | C | D | E | F | G |  |  |  |  |  |  | 79.850000 |
| 1,2 |  |  | C | D | E | F | G |  |  |  |  |  |  | 79.775000 |
| 14,2 |  |  |  | D | E | F | G |  |  |  |  |  |  | 79.700000 |
| 1,1 |  |  |  | D | E | F | G |  |  |  |  |  |  | 79.550000 |
| 3,2 |  |  |  | D | E | F | G |  |  |  |  |  |  | 79.350000 |
| 8,2 |  |  |  | D | E | F | G | H |  |  |  |  |  | 78.325000 |
| 8,1 |  |  |  |  | E | F | G | H | I |  |  |  |  | 77.950000 |
| 3,1 |  |  |  |  | E | F | G | H | I | J |  |  |  | 77.500000 |
| 13,1 |  |  |  |  | E | F | G | H | I | J |  |  |  | 77.200000 |
| 6,2 |  |  |  |  |  | F | G | H | I | J |  |  |  | 76.900000 |
| 11,2 |  |  |  |  |  | F | G | H | I | J | K |  |  | 76.825000 |
| 12,1 |  |  |  |  |  | F | G | H | I | J | K |  |  | 76.700000 |
| 14,1 |  |  |  |  |  |  | G | H | I | J | K | L |  | 76.300000 |
| 12,2 |  |  |  |  |  |  |  | H | I | J | K | L |  | 75.150000 |
| 10,2 |  |  |  |  |  |  |  |  | I | J | K | L | M | 74.250000 |
| 9,1 |  |  |  |  |  |  |  |  |  | J | K | L | M | 73.950000 |
| 4,2 |  |  |  |  |  |  |  |  |  |  | K | L | M | 73.000000 |
| 2,2 |  |  |  |  |  |  |  |  |  |  |  | L | M | 72.550000 |
| 13,2 |  |  |  |  |  |  |  |  |  |  |  |  | M | 70.550000 |

**ELECTROLYTE LEAKAGE**

| **Source** | **DF** | **Sum of Squares** | **Mean Square** | **F Ratio** |
| --- | --- | --- | --- | --- |
| Model | 27 | 4208.4074 | 155.867 | 27.1173 |
| Error | 84 | 482.8225 | 5.748 | **Prob > F** |
| C. Total | 111 | 4691.2299 |  | <.0001* |

| **Source** | **Nparm** | **DF** | **Sum of Squares** | **F Ratio** | **Prob > F** |
| --- | --- | --- | --- | --- | --- |
| Çeşit | 13 | 13 | 1117.7287 | 14.9584 | <.0001* |
| Soğuk | 1 | 1 | 1859.0151 | 323.4258 | <.0001* |
| Çeşit*Soğuk | 13 | 13 | 1231.6637 | 16.4832 | <.0001* |

| **Level** |  |  |  |  |  |  |  | **Least Sq Mean** |
| --- | --- | --- | --- | --- | --- | --- | --- | --- |
| 2 | A |  |  |  |  |  |  | 33.800000 |
| 3 | A |  |  |  |  |  |  | 33.612500 |
| 5 |  | B |  |  |  |  |  | 30.312500 |
| 6 |  | B | C |  |  |  |  | 29.812500 |
| 8 |  | B | C | D |  |  |  | 28.362500 |
| 13 |  | B | C | D |  |  |  | 28.312500 |
| 14 |  |  | C | D | E |  |  | 27.575000 |
| 1 |  |  |  | D | E |  |  | 27.050000 |
| 7 |  |  |  | D | E |  |  | 26.900000 |
| 4 |  |  |  | D | E |  |  | 26.475000 |
| 11 |  |  |  |  | E | F |  | 25.900000 |
| 10 |  |  |  |  |  | F | G | 23.837500 |
| 12 |  |  |  |  |  | F | G | 23.700000 |
| 9 |  |  |  |  |  |  | G | 23.362500 |

| **Level** |  |  | **Least Sq Mean** |
| --- | --- | --- | --- |
| 1 | A |  | 31.860714 |
| 2 |  | B | 23.712500 |

| **Level** |  |  |  |  |  |  |  |  | **Least Sq Mean** |
| --- | --- | --- | --- | --- | --- | --- | --- | --- | --- |
| 3,2 | A |  |  |  |  |  |  |  | 36.425000 |
| 1,1 | A | B |  |  |  |  |  |  | 34.800000 |
| 2,2 | A | B |  |  |  |  |  |  | 34.500000 |
| 6,1 | A | B |  |  |  |  |  |  | 34.500000 |
| 2,1 | A | B | C |  |  |  |  |  | 33.100000 |
| 7,1 |  | B | C |  |  |  |  |  | 32.350000 |
| 13,1 |  | B | C |  |  |  |  |  | 32.350000 |
| 12,1 |  | B | C |  |  |  |  |  | 32.300000 |
| 4,1 |  | B | C |  |  |  |  |  | 32.000000 |
| 9,1 |  | B | C |  |  |  |  |  | 31.750000 |
| 3,1 |  |  | C |  |  |  |  |  | 30.800000 |
| 5,1 |  |  | C |  |  |  |  |  | 30.750000 |
| 8,1 |  |  | C |  |  |  |  |  | 30.600000 |
| 10,1 |  |  | C |  |  |  |  |  | 30.600000 |
| 11,1 |  |  | C |  |  |  |  |  | 30.200000 |
| 14,1 |  |  | C |  |  |  |  |  | 29.950000 |
| 5,2 |  |  | C |  |  |  |  |  | 29.875000 |
| 8,2 |  |  |  | D |  |  |  |  | 26.125000 |
| 14,2 |  |  |  | D |  |  |  |  | 25.200000 |
| 6,2 |  |  |  | D |  |  |  |  | 25.125000 |
| 13,2 |  |  |  | D | E |  |  |  | 24.275000 |
| 11,2 |  |  |  |  | E | F |  |  | 21.600000 |
| 7,2 |  |  |  |  | E | F |  |  | 21.450000 |
| 4,2 |  |  |  |  | E | F |  |  | 20.950000 |
| 1,2 |  |  |  |  |  | F | G |  | 19.300000 |
| 10,2 |  |  |  |  |  |  | G | H | 17.075000 |
| 12,2 |  |  |  |  |  |  |  | H | 15.100000 |
| 9,2 |  |  |  |  |  |  |  | H | 14.975000 |
